# A Physiological Essay on the Thymus Gland

**Published:** 1845-07

**Authors:** 


### A Physiological Essay on the Thymus Gland. By *John Simon*, F.R.S. Fellow of the Royal College of Surgeons, Demonstrator of Anatomy in King's College, London, and Assistant Surgeon to the King's College Hospital. 4to. pp. 100. Renshaw, London, 1845.

We learn from the Preface, that this is the Essay for which the triennial prize of £300., arising from a sum of money bequeathed by the late Sir Astley Cooper, was awarded to Mr. Simon by the Physicians and Surgeons of Guy's Hospital. We sincerely congratulate the author upon being the first person to carry off the prize munificently instituted by our great surgeon for the encouragement of original investigations in physiology and surgery. This endowment is really a handsome and substantial reward, completely eclipsing the Jacksonian Prize, the paltry amount of which (£10.)* is barely sufficient to meet the expense of pen and paper. In one instance the successful author paid double the amount to an amanuensis for copying the essay, and ten times the sum has been expended on drawings to illustrate it. The inducements to prosecute arduous researches in anatomy and physiology are, in this country, few indeed. We may indulge a hope, therefore, that the liberal example of Sir Astley Cooper will be followed, and that those who have benefited largely by their profession, will evince the estimation in which they hold it, by encouraging their successors to devote their energies and abilities to the advancement of medical science. Let us not forget the words of Bacon :—" I hold every man a debtor to his profession ; from the which as men of course do seek to receive countenance and profit, so ought they of duty to endeavour themselves to be a help and ornament thereunto."

The author commences in Chapter I. with a Historical Introduction, which is concise, and exhibits much careful research. He observes that, the existence of the Thymus gland was probably first noticed in the Museum of Alexandria. Rufus, the earliest compiler, whose works comprise the discoveries of that celebrated school, mentions it as having already obtained a Greek name. Hippocrates had previously omitted it in his enumeration of the glands, and Aristotle on several occasions had left it unmentioned. Galen and Julius Pollux are the next writers after Rufus who mention this gland : the latter dismisses it in few words ; the former gives a more detailed account, and attributes to it the double office of defending the vena cava from the contact of the sternum, and of staying and supporting this large vessel with its branches. The same genuine physiologist was the first to notice the differences produced by age in the size of the gland. After briefly reviewing the progress of physiology and of structural anatomy during the sixteenth and seventeenth centuries, the author remarks :

" Simultaneous with this general progress in structural anatomy—and indeed

---

* This amount has within the last few years been increased by the College of Surgeons to twenty guineas.

forming part of it—was a better knowledge of the Thymus gland. Blasius had dissected it in a variety of animals; Bartholin, Horstius, Harvey, Deusingius, de Graaf, Diemerbroeck, and Munnicks, had noticed its fluid contents: Wharton and his followers had recognized its true structure sufficiently to arrange it with the conglomerate glands, and to compare it especially with the pancreas and parotid; its arteries and veins had been traced with tolerable exactness, and its nerves and lymphatics described, though not always with strict fidelity.

"With these additions to positive knowledge, an immense number of crude hypotheses are presented to us, and these have so much extended themselves towards the present time, that it will be convenient in reviewing them to follow each particular theory through its later developments, and to consider in one connected view the statements of its early and more modern supporters." P. 6.

The following passage will be read with interest.

"Glisson first originated an opinion which is interesting as being nearly the same as Sir Astley Cooper's;—quemadmodum mammæ lac infantulo foris præparant, sic Thymus liquorem nutritium intus suppeditaverit: Dr. Charleton adopted, a few years afterwards, the very words of this suggestion. The same views were taught by the celebrated Dionis in his demonstrations of Anatomy from 1673 to 1680, as also by St. Hilaire at the same period, and by Garengeot thirty years later. Puteus partially adopts the theory, and Ludovico Palliani quotes one of his colleagues as having just held a disputation (1758) concerning the Thymus, in which he maintained—'ei prædictam glandulam muneri inservire, ut ex integrâ sanguinis massâ substantiam quandam lacteam nutriendo fœtui necessariam secerneret.'

"From this time until the period of Sir Astley's work, the opinion seems to have been forgotten, and the terms in which he advances it lead me to believe that he was unconscious of agreement with the authorities just quoted. 'Is it not probable,' he asks, 'that the gland is designed to prepare a fluid, well fitted for the fœtal growth and nourishment, from the blood of the mother, before the birth of the fœtus, and consequently before chyle is formed from food; and this process continues for a short time after birth, the quantity of fluid secreted from the Thymus gradually declining as that of chylification becomes perfectly established?" P. 7.

If Sir Astley Cooper had been a better-informed anatomist, he might, in this instance, not only have done justice to his predecessors, but have saved himself some labour and fruitless speculation. It is unnecessary to follow our author closely in the account which he gives of the crude and often absurd theories which have been promulgated by different physiologists respecting the office of the Thymus gland. Among the speculations which refer it to the lymphatic system, occur the interesting researches of Hewson, and his discovery of the peculiar corpuscles which the gland contains.

"While examining its fluid with a glass of $\frac{1}{23}$ inch focus, he perceived 'a great number of small, white, solid particles, exactly resembling in size and shape the central particles in the vesicles of the blood, or such as are found in the fluid of the lymphatic glands.' To these last he had previously attributed the destiny of becoming nuclei for the vesicles of the blood; and he now surmised that the Thymus might be considered as an appendage to the conglobate glands, and participate in their function of forming the nuclei. He considered the probability of this to be the greater, 'from observing that the Thymus exists during the early period of life only, when these particles seem to be most wanted;' and he 'found reason for suspecting, that the lymphatic vessels were possibly the excretory ducts of the Thymus.' After a long period of neglect, Hewson's general theory of the relation of the lymph-corpuscles to the coloured particles of the blood, has received extraordinary confirmation, and almost universal acquiescence." P. 9.

That particular portion of it which concerns the Thymus seems to Mr. Simon, for many reasons, inadmissible; but it has lately obtained partial support from Bischoff, one of the most eminent German physiologists.

Among the most interesting and plausible hypotheses on our subject, must be accounted those which refer the activity of the Thymus to the quiescence of the lungs, and suppose that, more or less directly, it fulfils in fœtal life the later functions of these organs, either in oxydizing or in decarbonating the blood. Our author remarks :—

"I shall have occasion hereafter to refer again to these views of the relation of the Thymus to the respiratory organs, and shall now merely observe that the arguments used against them by Becker and Haugsted are quite unanswerable; being founded on the fact that the chief development of the gland is not during the period of uterine life, nor the commencement of its decay simultaneous with birth; its activity, in other words, is *not* greatest during the quiescence of the lungs; although then, according to the opinions quoted, there would be the chief, or only, necessity for its decarbonizing action on the blood." P. 11.

The most important recent contributions to the general anatomy of the Thymus are to be traced in the monographs of Meckel, Lucæ, Haugsted and Cooper. Meckel carefully considers the circumstances which determine a greater size or longer persistence of the Thymus ; investigates it especially in rodent and marine mammalia ; notices for the first time, in certain birds, an organ which he believes to be analogous to that under consideration, and quotes numerous instances of disease affecting it in the human subject.

"Lucæ's investigation of the Thymus was anatomical, and chiefly regarded its minute construction. He recognized its cells and their arrangement around the lobular cavity, and distinguished the former as secretory, the latter as receptacular ; but he erroneously considered each cell as an acinus containing within itself a plexus of blood-vessels. Tiedemann more correctly explains the true relation of these anatomical elements : he describes each lobule as formed of many cells, round which a vascular network is expanded, and the cells as all communicating with the common cavity of the lobule.

"Sir Astley Cooper's work finally fixed this view of the structure of the gland as completely as could be done without the use of the microscope. By means of very fine and successful injections, he filled the minutest cavities of the organ, and displayed the relations of each to the common cavity of the whole." P. 14.

Sir Astley Cooper's anatomical conclusions are so well known that it is needless to quote them. In the same year with this work appeared the far different one of Dr. Haugsted. Mr. Simon states, "It is less remarkable for anatomical than for literary research, but in this respect is unrivalled; every author who has written on the subject is made tributary to its pages, and it is scarcely possible to improve on his selection as regards either the number or pertinence of the quotations ; his criticism of the various opinions is likewise sound and perspicuous. To our knowledge of the anatomy of the gland he contributed little or nothing; in comparative anatomy he made several examinations, though not always with the happiest results. Perhaps the chief physiological merit in his work, is the prominence he gives to the fact of the increase of the Thymus after birth; for, although Verheyen, Palfyn, and others, had cursorily noticed this circumstance, it had not obtained general attention; and men had continued arguing and inventing hypotheses for the Thymus as a fœtal organ, in ignorance of the essential error which must infect every theory so founded. Haugsted established, beyond the possibility of doubt, that the Thymus does not attain its greatest development, either absolute or proportional, until after a considerable interval from birth."

Our author observes:—

"With the two last-mentioned writers terminates the literature of our subject so far as concerns that elder school of anatomy, which contented itself with an almost exclusive study of relative position and outward form. To that school of descriptive anatomy we owe the greatest obligations. From the time of Galen to our own, it has been intimately connected with the progress and practical perfection of the healing art, and has included all the greatest masters of our profession; it has produced important physiological discoveries, has created our means of distinguishing disease, and laid the foundations of scientific medicine. It may at length be thought to have exhausted its materials, and fulfilled its task." P. 16.

Mr. Simon next gives a masterly sketch of the labours of the new school of structural anatomy, doing ample justice to the researches of Schwann, and acknowledging the merit of others, on the continent and in this country, who have successfully cultivated this branch of science. He observes, in conclusion, that no attempt has hitherto been made, amid the new school of physiology, to expound the structure or inquire the uses of the glands without ducts, in the cautious spirit of philosophical generalization. Yet there do seem to exist in the present day nearly all the elements so long wanted for resolving the problem which is the subject of the present essay: the means, on the one hand, for correctly interpreting the ultimate anatomy of organs; and, on the other, for correctly expressing the nature of their functions in the terms of chemical science. Under these advantages, we hope that we shall not be disappointed with the results of the difficult investigation undertaken by the author, and which have gained for him the Cooper prize.

The subject of the Second Chapter is the Development of the Thymus. The author very properly omits the details of exterior anatomy—topics already fully exhausted—*the functions and intimate structure of the gland* being the peculiar subject-matter of his essay. The first appearance and early growth of the Thymus are points of much interest in its history. Mr. Simon's investigations have been chiefly conducted on the embryos of swine and oxen.

"The earliest form, in which I have discovered it, has been that of a simple tube; lying, in the animals I have mentioned, along the carotid vessels, and surrounded by the faint indications of nascent areolar tissue. The contents of the tube are seen (with a magnifying power of 400 diameters) to be granular and dotted, but do not as yet shew distinct corpuscles. Its figure is defined by the abrupt outline of the membrane, which constitutes the wall,—an exquisitely delicate, transparent, homogeneous tunic, presenting at regular intervals slight elongated thickenings of its substance." P. 20.

Mr. Simon believes, for various reasons, that the first rudiments of the gland would be found in a series of primordial cells, arranged in a line along the cervical vessels and pericardium, and coalescing on each side of the neck to form the tube he has described. This mode of origin would be in strict accordance with analogy, and would explain the peculiar thickenings which are found in the wall of the tube.

"The second stage in the progress of development is in analogy with the mode of growth attributed to true glands: the tube bulges at certain points of its length, on one side or the other, and gives origin to diverticula or follicles, which maintain their connexion with its cavity. These follicles have precisely the same contexture as the parent tube, of which in fact they are mere evolutions; they are bounded by the same delicate tissue as constitutes its wall; they contain, too, the same material, in which may now be seen the peculiar dotted corpuscles hereafter to be described." P. 22.

The form of these growths from the tube "in the present stage, is that of larger or smaller segments of a circle; sometimes projecting but little beyond the general line of the tube, sometimes exactly hemispherical, but always tending in their growth to become peninsular, and to retain their connexion with the main canal by means of a narrow isthmus of communication. Thus in the portions of the gland, where they are most thronged together, they may be seen in profile forming a series of flask-like appendages to the tube."

"The third distinct step of advancing development is made by the commencement of ramification in the follicles. This process usually begins when the follicle has attained the shape of three-fourths of a sphere, or somewhat later; and occurs without any elongation of the isthmus connecting the follicle with the main canal; so that these secondary and tertiary extensions of the cavity do not possess the tubular structure in so marked a degree, as the axis from which they spring." We shall not follow the author in his minute account of the formation of the follicles, as our readers would not comprehend it without the diagrams which illustrate the original. In concluding the description, he states: "By the extension of this process of follicular growth to all portions of the gland successively,—by the repetition on each new crop of follicles of the same acts of development and ramification,—and by the continued molecular increase which pervades the entire substance uniformly, as the means of interstitial growth, the Thymus attains the bulk and the complexity of structure which distinguish it in the mature fœtus. There is no change whatever in the nature of the phænomena: the later steps are exact iterations of the first; the type of development is established by the earliest bulging of the primary tube—by the first vesicle that buds from its side. That type essentially consists in the *lateral growth of branching diverticula from a central tubular axis*. And as the extremities of these diverticula, like the follicle whence they first sprang, tend always to assume the vesicular shape, and to represent large segments of spheres, the permanent structure of the gland, no less than its mode of development, may be named *tubulo-vesicular*."

We here find explained an error committed by Sir Astley Cooper in describing a cavity to which he gave the name of *reservoir*. This is attributed to an undue distention of the interior of the gland.

Mr. Simon justly assumes that the function of the Thymus must assuredly be most energetic at the time when its size is largest, and, having incontestibly proved, by facts adduced by Haugsted and himself, that the principal development of the gland is at a period considerably later than birth, he at once extinguishes all those theories which connect the uses of the organ with the peculiarities of fœtal organization and fœtal life. He gives a table of 64 experiments, exhibiting the weight of the Thymus in various animals and in the human subject at different periods, all of which strictly agree in their testimony to the increase of the Thymus after birth. The general and rough results of the examinations of the human subject seem to be the following:

"First, During the period next succeeding birth, the activity of the Thymus is remakable; it increases considerably in size, becomes turgid with secretion, and its specific gravity is lowered by the greater fluidity of its contents. The first growth is far out of ratio to the general increase of the body, but gradually subsides into a stage of less activity, which merely suffices to maintain the proportion so acquired.

"Secondly, During several months it continues to increase at a diminished rate, and merely in proportion to the general growth of the body; its further enlargement ceases about two years after birth.

"Thirdly, From this time, during a very variable number of years, it remains stationary; and, supposing the individual to be adequately nourished, gradually assumes the structure of fat. This stage, in which the bulk remains unaltered, but the texture changes so curiously, extends perhaps in the largest number of healthy individuals to the 8th, 9th, 10th, 11th or even 12th year of life; but it cannot be restricted even to these loose limits, for some years later the gland will often appear to have undergone no diminution in size. The intimate nature of its interesting structural change will be explained in connexion with the general morphology of the gland.

"Fourthly, The duration of its decay, and the epoch of its entire vanishing, are still more uncertain. About puberty it seems, in most cases, to suffer its chief loss of substance, and to be reduced to a vestigiary form; but still for many years its flat and emaciated lobes may often be dissected from the pericardium, and shewn as a connected body. Distinct remnants of the gland may generally be exhibited in subjects of from twenty to twenty-five years of age; but beyond the latter time it is unusual to distinguish any positive traces of its existence amid the areolar tissue of the mediastinum. There are exceptions to this rule. I have sometimes discerned faint remnants of its form in subjects up to thirty years old; and Meckel and Haugsted quote various instances of its alleged persistence to a much later period. In several of these last-mentioned cases the gland was evidently the seat of the disease; and in others its abnormal continuance seemed associated with other morbid affections, chiefly of the respiratory organs; such irregularities need not interfere with our calculation of the usual date of its disappearance." P. 32.

In Chapter III, the author considers the Mature Structure of the Gland and Nature of its Secretion. Its structure presents for consideration the following particulars:—First, The arrangement of its cavity; secondly, the texture of the walls of that cavity; thirdly, the nature of its contents; fourthly, its means of vascular and nervous organization.

In closely examining the surface of the Thymus, after the removal of all vessels and areolar tissue, we observe that it is divided into a vast number of apparently distinct portions, measuring in diameter from half a line to almost two lines. These minute parts are recognised as membranous cavities, and are the terminal vesicles of the gland: on dissection, it is possible, without disturbing any natural union of parts, to resolve the structure of the gland into masses ranged round an axis. "Each mass constitutes a sort of cone of glandular substance;—its apex pointing to the axis, or mesial line, of the gland; its base directed to the surface, where it presents its innumerable vesicles; while its intermediate part contains those successive branchings of the follicle, which terminate superficially in the vesicular form."

Mr. Simon describes the texture of the walls of the cavity as a material of the most exquisite delicacy, and as not exceeding $\frac{1}{15000}$ of an inch in diameter. It is transparent and quite homogeneous. A close capillary network furnishing the materials for secretion, lies in intimate contact with its outer surface, and is adapted to its various irregular inflexions.

"This exquisitely attenuated, but tough and elastic, membrane is the only proper wall of the glandular cavities. It is strengthened on its exterior surface by an expansion of areolar tissue, which penetrates into every interval, and invests every part of the organ. This is of extreme fineness where it clothes the vesicles and can only be adequately examined by the aid of the microscope. Intermixed with it may be seen a small proportion of elastic tissue, in the form of very delicate straight fibrillæ crossing the surface of the vesicles in various directions, so as to form a network with wide meshes. It is probably these fibrillæ that Dr. Pappenheim has mistaken for organic nervous fibres." P. 35.

The contents of the cavities of the Thymus consist of a fluid, in which (as Hewson discovered) an immense multitude of microscopical corpuscles float. These corpuscles are found to be circular discs of nearly the same size as the coloured particles of the blood. They are generally flat and circular, but a large number may be found in every examination deviating more or less from the discord shape, and tending to become globular, or to assume irregular outlines. They have certain characteristic markings of their substance in the form of a variable number of minute dottings. These are supposed to be the minutest molecules of fat in combination with the solid albumen or fibrin, which constitutes the bulk of the corpuscle.

The following is an analysis of the Thymus, given by Mr. Simon, it being impossible to obtain the secretion apart from the secretory structure.

"*Analysis of Thymus of Calf about three months old.*

| | |
|---|---|
| Water .. .. .. .. .. .. | 77·20 |
| Fibrin, gelatinous tissue, and traces of fat .. | 12·72 |
| A substance between Albumen and Casein .. | 4·13 |
| Watery extract .. .. .. .. .. | 3·80 |
| Salts, principally phosphates of Soda and Lime.. | 2.15 |
| | 100·00" |

The results of this examination, and of those of Sir A. Cooper's and Morin's, agree in a particular of extreme importance; they conclusively demonstrate, that no theory for the use of the Thymus can be just, which involves the supposition of its secreting and containing highly-carbonated matters, through the period of uterine life.

"Tiedemann, Arnold and other almost equally great authorities have argued, that the activity of the Thymus is in inverse proportion to that of the lung, and that, during the quiescence of this organ, it effects for the embryo a kind of vicarious respiration by separating a carbonaceous product from the blood. Chemical analysis suffices to display the instability of this hypothesis; it shews that the Thymus, in the period of its highest activity, instead of being surcharged with carbon, in reality contains no more of that element, than may be found in blood or muscle.*" P. 36.

Mr. Simon states that the actual and ultimate nature of the secretion of the Thymus is expressed, as nearly as may be, by the formula of Protein; in other words, it is identical with the common material of organic nourishment. And perhaps the greatest positive service, which chemistry can render to the present investigation, is thus to shew us that we may securely dispense with the special phrases of the laboratory, and may venture, in general physiological language, to describe the *secretion of the Thymus gland in the young animal as* NUTRIENT MATTER.

After mentioning the general courses of the arteries and veins, Mr. Simon describes the nature of their capillary terminations. These he has frequently injected; and so abundant is their distribution, that, in a case of successful injection, the whole organ is deeply coloured with the material employed.

" A specimen of this kind, examined under the microscope, shews the injected capillary network to be of the completest description. It is so arranged as to include each individual vesicle within a vascular capsule; the capillaries are closely applied upon the transparent texture (*limitary membrane*), which bounds the cavities, and so exceedingly dense is their network, that the meshes are of even less diameter than the vessels themselves. Every portion of the glandular substance is thus exposed in the completest manner, and at every point of its surface, to the penetration of the fluid ingredients of the blood." P. 38.

The author is unable to offer any satisfactory account of the real arrangement of the lymphatics. He argues that they do not fulfil the office of carrying off the secretion in the manner of excretory ducts; that here, as elsewhere, they serve only to appropriate and convey, in their own mysterious manner, certain interstitial superfluities of the nutritive process. The nervous supply of the gland is mainly derived from the plexus, which surrounds the first part of the subclavian artery, and which has its chief origin from the *inferior and middle cervical ganglia.* A second source of supply is the cardiac branch of the *pneumogastric,* which gives on each side a minute filament to the superior part of the gland.

In Chapter IV, the author treats of the Comparative Anatomy of the Thymus. He states that he has devoted great labour to this subject, and has been fortunate in obtaining the means of dissecting many rare animals.

---

* As may be seen in comparing the subjoined ultimate analyses of the organic portions of blood, muscle, and thymus, viz.—

| | Flesh. | Blood. | Thymus. |
|---|---|---|---|
| Carbon .. .. .. | 54·12 | 54·20 | 54·02 |
| Hydrogen .. .. | 7·89 | 7·65 | 8·12 |
| Nitrogen .. .. | 15·67 | 15·73 | 13·42 |
| Oxygen .. .. .. | 22·32 | 22·12 | 24·44 |

The general results of his dissections may be stated as the discovery, that *the Thymus gland belongs, without exception, to all animals breathing by lungs, and to no others.* We regret that we cannot find space to accompany our author in the admirable account which he has given of his numerous and various dissections. His descriptions are concise and perspicuous, and abundantly illustrated by well-executed wood-cuts. This important section of his subject will be read and studied by all physiologists and comparative anatomists. He completely overturns the prevailing doctrine that the Thymus is an organ strictly limited to the class of mammalia, and peculiarly adapted to their organization and mode of life. We can only quote the chief results of this comparative investigation.

"1. The *presence of the gland* is co-extensive with pulmonary respiration. 2. Its *shape and position* are variable and unimportant. 3. Its *size and duration* are, generally speaking, in proportion to the habitual or periodical inactivity of the animal. 4. Where it remains as a persistent organ, it is usually but one of several means for the accumulation of nutritive material: its continuance, under such circumstances, is generally accompanied—though, in some instances, superseded—by a peculiar accessory contrivance, the *fat-body*." P. 64.

In Chapter V. the author treats of the Morphology of the Thymus. This gland has long been associated in anatomical teaching with certain other bodies of equally obscure function; namely, with the spleen, the supra-renal capsules, and the thyroid body. We may naturally inquire, whether this classification has been made in the light of ostensible affinities; whether the several members of the order so constituted are actually bound together by physiological relationship, or merely dwell side by side as joint tenants of a common obscurity. After alluding to the opinion of Henle, who adopts the latter belief, Mr. Simon states that the results of his inquiries enable him "to affirm with confidence, that they do constitute a strictly natural family; that, in all the elements of their composition, they admit of detailed comparison with the so-called *true glands* of the body, forming a series parallel with theirs; and that the title of *glands without ducts*, for a long while vaguely applied to them, rightly expresses these homological relations."

Before advancing to the consideration of what is peculiar to the Thymus, he proceeds to demonstrate the characteristics of the class to which it belongs; and while justifying the constitution of this gland, he endeavours to illustrate the points of similarity and of difference, by which the organs included in it are related to the true glands; the points, in short, by virtue of which the function of these bodies may be considered as forming part of the general problem of secretion. After alluding to the well-known view of Müller as to the nature of a gland, and to the researches of Purkinje, Valentin, Henle, and Schwann, on its essential anatomy and organic means of secretion, Mr. Simon gives his own views on this difficult subject.

"The organic means for secretion, as illustrated in the anatomical structure of true glands, may be stated to consist in an arrangement for the growth of deciduous cells, in close relation with blood-vessels on the one hand, and with an evacuant channel on the other.

"All the chief acts of the vital economy are carried on by the medium and instrumentality of cells: in evidence of which fact it will be sufficient to cite the process of growth in its various modifications,—whether as seen in the development of the embryo, or in the repairing of injured textures, or in the organization of morbid products. Further, by the proportion in which these microscopical elements (or their rudiments, or their reliques) are present, we are enabled to measure the functional activity of any particular organ; their plenteousness and constancy are direct indications of abundant organic change—of life active in the part."

"In examining under the microscope a thin section of any one of the true glands, we find its bulk mainly consisting of cells, or their rudiments, in the losest possible aggregation; nowhere in the adult body do we find greater evidence of nutritive activity than in such a specimen; it is as obviously a growing structure as if it had formed part of an embryo. And when, after contemplating the important functions discharged by cells in other organs of the body, we turn to consider the use and object of their extreme preponderance in glandular structure, we are impelled to believe their essential connexion with the processes here effected. Every analogy leads us to anticipate that here, as elsewhere, they should be the media of organic change,—that their growth should inseparably identify itself with the manifestation of whatsoever specific materials it may be the function of their particular gland to eliminate from the system." P. 67.

The preceding observations are illustrated by a few unquestionable examples. They are so interesting, that, notwithstanding the length to which our review has extended, we cannot forbear quoting them.

"In the liver, it is quite certain that the bile is for a period contained within the cavity of the cells. Henle, in confirmation of Hallmann's observation, states that he has seen these corpuscles of a yellowish tint, and ascribes that hue to the colouring matter of their secretion. But the natural appearance of the liver-cell is liable to certain exaggerations, which, though they originate in disease, are yet serviceable additions to the knowledge derived from healthy structure. For instance: the frequency of enlargement of the liver in cases of pulmonary disease, and its disposition to compensate for defective function at the lung by increased activity of its own secretion, had long been known; Louis had directed attention to the remarkable number of instances in which its fatty degeneration is found coincident with phthisis; and it was a natural step in pathological reasoning to suppose,—as the two organs have a certain analogy of chemical action,—that this morbid development of fat in the liver might have reference to the superabundance of carbon in the system, which the diseased lung could no longer eliminate,—that it might be a sort of vicarious action. It is now known that the actual seat of this fatty deposit is within the cells of the gland, that there is in each cell a morbid increase of the oily matter, which it should naturally contain; and it is argued, with great probability, that these minute elements, so surcharged with their compound of carbon, are but the ordinary channels of health, and that the fat is but an exaggerated secretion of the gland, here surprised in its very transit from the system.

"Still more conclusive is the evidence furnished by the following fact, as it relates to changes which have a merely mechanical origin. In cirrhosis, the essential primary disease is an inflammatory action, under the influence of which a quantity of coagulable lymph is poured into the interstices of the vessels and ducts; and, as this product of inflammation becomes organised, it contracts very closely, and surrounds with a dense capsule various isolated portions of the hepatic substance, or forms tough septa and constrictions within the liver and on its surface. By the condensation of this adventitious material, and by its pressure on the normal elements of the gland, there are produced various secondary results, which depend on mechanical obstruction; for example, partial atrophy of some spots with apparent hypertrophy of others; or again, jaundice, or ascites.

Under these circumstances (probably where the strangulation has especially told on some small duct, and has obstructed or obliterated it) we find certain circumscribed masses of the gland coloured with the deepest yellow, from intense biliary congestion. These parts will invariably present the microscopic appearance to which I wish to refer; namely, the ultimate cells are identified as the seat of this deep ochrous colouring,—they are gorged with bile. And, as this phenomenon cannot be ascribed to the physical cause of imbibition, (for it always prevails first and chiefly in the interior of the cell, and about its nucleus,) there appears no other possible explanation of its occurrence, than the theory here defended,—that the secretions of glands first manifest themselves in connexion with cells, or with their germs,—that the secretory process in glands is one with the cell-growth of their parenchyma.

"In certain other glands I have likewise discovered a circumstance, which appears even more conclusive than that last mentioned, as to the point in question; viz., the occurrence within a nucleated cell-membrane of solid saline materials, corresponding to those of the secretion. In the urine of fishes, for example, and in the thyroid secretion of many animals, microscopical crystals frequently occur in considerable quantities; and, even where the crystalline form is incomplete, the same peculiar product may be recognised by its dioptric qualities, as its minute masses float in the fluid. In several instances, while examining the kidneys or thyroid gland with the microscope, I have noticed that certain of the cells (unquestionable cells, with complete membranes and distinct dotted nuclei) have been distinguished from those in their vicinity, by the fact of their contents possessing the same refractive properties as the floating particles of the secretion; often, too, I have succeeded in distinctly recognising a crystalline arrangement in these inorganic contents of a cell." P. 69.

Mr. Simon remarks, that there are many reasons for believing that the so-called nucleus, or *cytoblast,* of a cell, is its essential part, and capable by itself of fulfilling the entire functions which have been generally ascribed to the wall of the complete cell; and it is highly important for the physiological understanding of the glands without ducts, that the grounds of this belief should be examined.

"It appears that in the development of secretory cells, there are the following steps: First, the formation of the nuclei; Secondly, the deposition of material around them; which step seems the first evidence of their peculiar function; Thirdly, the isolation of this material by the growth of a membrane about it,—in other words, the completion of a cell, which has now all its elements—nucleus, membrane, and contents; Fourthly, a stage of apparent quiescence, during which the specific contents of the cell are probably either increased in quantity, or brought to greater concentration; a stage, in one word, of ripening: Fifthly, the falling of the cell with its contained material, in the form of excretion.

"Now, in certain cases (and these bring us very near to the habitual state of the glands without ducts), it seems that the third stage of this process is absent, that no cell-membrane is formed, that the nucleus, with the material developed round it, constitutes the sole physical evidence of activity in the part. Indeed, in all glands this stage appears far less complete than in other organs of the body; in most it seems an exception, rather than the rule, to find the cell-membrane perfectly and definitely formed; the liver is the chief—if not the only—instance to the contrary. Moreover, where we are able to trace the products of secretion actually within a cell (as in the above-quoted instance of cirrhosis of the liver) we find them either exclusively, or at least with a very marked predominance, accumulated in that portion which corresponds to the nucleus: as though this corpuscle were the true centre of attraction, and the cell-membrane only the boundary, or passive recipient, of the matter to be excreted.

"These considerations and—still more forcibly—various illustrations which may be gathered from the history of the suppurative process and from other pathological phenomena, lead us to the following conclusions: viz. (1) that the cell-membrane—whether perhaps it exert any specific vital influence on the matter with which it has contact, or merely serve for the mechanical isolation of its contents—must be viewed, at least in the secretory process, as a secondary and inessential formation: (2) that its existence, in nutrition generally, bears relation to the rate and perfectness of the cell-growth, and to the permanence of the organic combination effected in that process; that it will not occur where the particular nutritive act is ill-supported by the economy, where it is either absolutely or proportionally accelerated, or where (as in instances presently to be specified) the peculiar functions which have their centre in the nucleus, are exerted for a short time only, and for temporary purposes: (3) that (in respect of secreting organs particularly) the nucleus—from its constancy, from the priority of its formation, and from the peculiar arrangement which the secreted matters assume in reference to it—must be considered as the characteristic and essential part of the apparatus, not requiring the completion of a cell in order to the performance of its functions; and (4), that the act of secretion, though essentially homologous with ordinary molecular nutrition, is peculiarly prone, in various cases and for various reasons, to exhibit its process of cell-growth in a low, and as it were, aborted form.

"When I state that this lower condition of cell-development is the one usually manifested in the structure of the glands without ducts, and especially in the Thymus, it will not, I trust, be thought that I have wandered from the proper limits of my subject in the foregoing argument; the application of which will follow presently." P. 70.

We have felt at a loss to abridge the preceding particulars, so as to give a clear and connected view of the results of Mr. Simon's researches. He next traces the presence and arrangement of another, nearly universal tissue.

"If we examine, with a magnifying power of 300-400 diameters, the structure of a true gland, e. g., of the pancreas or kidney, we find that its assimilating cells are included within a simple continuous tunic; and, as we extend our inquiry, we learn that varieties in the arrangement of this membrane determine the several shapes affected in the ultimate structure of glands. Thus, whether we examine the botryoid vesicles of the salivary glands, the bulging tubules of the stomach, the simple follicles of the intestine, or the long windings and uniform calibre of the urinary and spermatic canals, we observe the outline of each various form to be marked and limited by the homogeneous membrane referred to.

"It is not peculiar to the true glands, but belongs equally to those without ducts; although it has been almost overlooked in their structure, and never correctly described. Nor is it even distinctive of the generic anatomy of glands; for a tissue identical with it is seen bounding the fasciculi of voluntary muscle, and the tubules of the nervous substance, as likewise occurring in various other situations. In the mucous and vascular systems it has been named *tunica propria*, or *basement tissue;* in the structure of muscle, *sarcolemma;* in that of nerves, *tubular membrane* or *neurilemma;* but, in each case, its anatomical relations are the same; it is, in each, the *barrier between nutrient vessels and the products of nutrition,*—a barrier that serves to support and to circumscribe the latter, but yet affords ready transit to the materials of constant renovation supplied by the former. Its functions and physical characters are strictly identical in all its various positions, whether it be seen as the single tunic of a capillary blood-vessel, or as bounding the ultimate structure of muscle, nerve, or gland; I have therefore ventured to apply to it a name which suits it equally in all these relations, and have, throughout this essay, spoken of it as the LIMITARY MEMBRANE.

"It extends, with more or less development, into every organ with deciduous cells, and constitutes in all (with certain rare but striking exceptions) a definite, but permeable, wall between the capillary blood-vessels on the one hand, and the assimilative cells, or cytoblasts, on the other. On its one surface, is the slow and equable circulation of blood through the finest net-work of capillaries; on its other, there advances the constant function of cell-growth, as I have above described it; while, intermediately, its own delicate tissue imbibes and is traversed by the liquor sanguinis, furnishing materials for the secretory process." P. 72.

In order to corroborate his theory of the use of the *limitary membrane* being merely mechanical, Mr. Simon refers to three remarkable instances of its absence, viz. in the liver and kidney amongst the true glands, and in the spleen, amongst the corresponding group of glands without ducts, and he endeavours to show that, in each instance cited, its absence appears to depend on some peculiar necessity in the organ, requiring that the access of liquor sanguinis to the glandular cells should be facilitated in the extremest degree. The opinion thus advanced is further strengthened by illustrations drawn from comparative anatomy.

In reference to the vascular organization of the true glands, it is remarked, that the larger arteries and veins follow no common law. That which is essential to function seems rather to be contingent on the nature of their ultimate, capillary distribution. In examining this we invariably find it to be such as may most effectively and uniformly diffuse the materials of nutrition amid the characteristic structures of the organ: it is a network of the extremest delicacy and closest woof, cast round about the ultimate tissues of the gland, and divided by the limitary membrane alone from the cells or cytoblasts which it serves to nourish. Since the "capillary blood-vessels are composed but of the thinnest homogeneous tissue, it is clear that their walls must be saturated, and freely and perpetually permeated by the fluid parts of the circulating blood; and since the same physical property of imbibition is possessed likewise by the limitary membrane, which divides the vascular network in question from the peculiar cells of the glandular parenchyma, we may justly consider that these last lie in an atmosphere of liquor sanguinis, and are developed in it, as their blastema." We regret that we are unable for want of space to follow our author in the further development of his views on the process of secretion and of the cell-theory. The following remarks, however, towards the conclusion, made in the true spirit of a philosophical inquirer, are so admirably expressed, that we cannot deny ourselves the pleasure of quoting them.

"In arriving at this point—the specific nutrition of gland-cells—we are as far as mere Anatomy, or *Statical Physiology*, can conduct us; yet how distant still from understanding the actual nature, the actual mystery, of the process! Doubtlessly it is a great generalisation—one that fills the mind of the physiologist with a sense of beautiful harmony—to find, as has been found, that the nutritive and secretory processes are essentially one; their organic instruments alike; their traceable steps parallel. But let not this brilliant discovery be misunderstood, or misapplied; the law developed in it is morphetical, and this merely. Whether we are occupied with the large, or with the little; whether we collect the flowing secretions from divided ureters and gall-ducts, or—armed with all optical resources, and standing on the very confines of the visible world—view the first molecules of bile and of the lithates, as they gather in their respective cells; equally in either case the pursuit is mere anatomy; equally in either case we are in the domain of form and phenomenon, not in that of power and law. With respect to secretion, the microscope has only shewn us in ultimate detail what for ages had been known in its broader features; it has but revealed of the molecules that which was known of the masses. Physiological difficulties are not contingent on size: 'Why does the liver-cell contain oil-globules and yellow matter, rather than urea and the lithates?' is a riddle involving the same speculations and requiring the same answer, as that plainer question of our forefathers, 'Why does the liver secrete bile rather than urine?' For the solution of these doubts, it is in vain that we scrutinise the lifeless molecules and mechanism of the dissected body. The glands are too essentially alike for us to venture on ascribing to their fancied diversities of affinity and filtration the manifoldness of their several products." P. 77.

Having endeavoured clearly to set forth the essential anatomy of true glands, Mr. Simon, in pursuance of his scheme, proceeds to apply the standard of comparison so obtained to the interpretation of the structure and functions of the Thymus. The special distinction of the *glands without ducts,* is found (as their name implies) in the circumstance of their lacking an apparatus of excretion; while their generic affinity to the true glands consists in their possession of all the organic means for secreting—though only into their own closed cavities. Mr. Simon next analyses these means for secretion, in the same order as was adopted in describing the true glands. He describes the cells as " discoid, and of about the size of blood-corpuscles (in the spleen and supra-renal capsules somewhat larger, in the thyroid and Thymus rather smaller), but possessing less thickness. They have the general appearance and physical properties of those dotted discs which we encounter, as the nuclei of cells, in true glands and other structures of the body; and they are, as I shall shew beyond doubt, identical with them in function." An account is then given of their differential characters in the thyroid body, supra-renal glands, spleen, and Thymus. The *limitary membrane* admits of demonstration in the glands without ducts, co-extensively with its existence in the true glands. The author gives a particular description of it in the thyroid body, and supra-renal glands, and notices its absence in the *Malpighian bodies of the spleen,* which is explained in a very interesting manner.

" In approaching the limitary tissue of the *Thymus,* the reader will anticipate my statement, that this it is which we have already noticed on several occasions. He will identify it with that simple diaphonous membrane, which, in early embryonic life, formed the primary tube of the nascent gland; he will remember how, by bulging and branching, it formed a ramified follicular system; and how it has been seen to constitute in the mature organ the exact boundary of the parenchyma." P. 82.

The *ultimate vascular organisation* of the glands without ducts is the same as that of the true glands; it consists in the distribution of a perfect capillary network around the assimilative elements. In the Thymus, the distribution of the plexus around the terminal vesicles is such, that it could hardly be distinguished from the secretory network of the pancreas.

The author subjoins a series of propositions as general results of the arguments on the subject of secretion.

"1. The *phenomenon of secretion* essentially consists in the manifestation of particular products around organic centres or nuclei, which develop themselves in the effused plasma of the blood, and which, from their actual or potential relation to cells, are called cytoblasts.

"2. The *cytoblasts* bear no varying relation to the nature of the particular secretion, but are in all instances of substantially the same composition as the liquor sanguinis from which they are engendered, and of which they may be considered a mere solidification.

"3. The *materials of secretion*, if solid, arrange themselves in almost impalpable molecules around the several cytoblasts; if fluid they constitute a common medium, in which the cytoblasts float.

"4. The *completion of a cell*, for the isolation of so much of the secreted product as is collected round each cytoblast, is a very frequent secondary process. In the true glands it is very frequent; in those without ducts exceptional. The steps of its development, and the circumstances regulating its existence, may be illustrated seriatim: viz.—

"α. In the Malpighian glandules of the spleen,—where the secretion is fluid and can but rarely be detected by the microscope in a molecular form, where the nutrition fluctuates from hour to hour, and where the assimilative affinities are therefore exerted with equal intensity only for the shortest periods,—a cell rarely, if ever, exists.

"β. In the Thymus,—where also during infancy, the secretion is fluid, and where (as I shall presently further illustrate) the assimilative acts probably vary in intensity over short and frequent periods, the persistence of cytoblasts without cells seems at first sight equally regular. And such is actually the case during the time of the gland's most active function; but when it becomes comparatively quiescent, or when (as in many reptiles and some mammalia) it assumes the characters of a permanent organ, it will be found that its cytoblasts have undergone their complete development, and become nuclei of the fat-cells which are formed within the limitary membrane.

"γ. In the tubes of the supra-renal glands, where the product is solid, there is constant opportunity of observing that transitional stage, in which the secreted matters are closely aggregated in a molecular form around the several cytoblasts; here, too, the completion of a cell is frequent.

"δ. In the vesicles of the thyroid gland, owing to the fluidity of the secretion, no intermediate stage of cell-growth can be seen; but cells, taking the characteristic cytoblasts of the organ for their nuclei, are often developed, and may be seen to contain a fluid of the same nature as that wherein they float.

"ε. Where the secretion of mucous surfaces is hurried by irritation, the bulk of it is found to consist of floating cytoblasts, which would else have been nuclei of epithelial cells; and the suppurative process, which is a similar abortion of cell-growth, indicates the operation of like influences in its origin.

"ζ. In the true glands, the formation of a complete cell is at once recognised as the regular type for secretory nutrition: The exceptions are very frequent, yet they admit of ready explanation on principles here stated; they are fallings short of the mature process, by the omission only of that which is in-essential; they are instances of unfinished development, under circumstances of local excitement or general ill-supply.

"5. The *vascular apparatus* is nearly identical in all organs of secretion; its main requisite being a distribution of blood in such delicate vessels, as may readily permit the transudation of its fluid ingredients.

"6. In most instances the cytoblasts are formed, and the particular products manifested on the surface of a *limitary membrane*, which divides them from immediate contact with the capillaries: but this tissue is absent under circumstances which exclude the possibility of its being essential to the process of secretion. It seems entirely subservient to the mechanical office of support: it is found in the true glands to be prolonged into the excretory apparatus, in those without ducts to be folded into closed tubes or vesicles; so as in the latter case to retain, in the former to conduct, the secretion.

"7. A distinction, implied in this differing arrangement of the limitary tissue, divides the glands into the two classes, which I have compared and contrasted. The specific difference of the smaller group consists in their not excreting from the body those materials, which are shed into them from the blood. *Their secretion is into closed cavities.*

"8. The glands without ducts *vary in activity* according to varying circumstances; sometimes their cavities are distended, sometimes nearly empty. In the latter case, that, which they had withdrawn from the blood, must again have passed into it.

"9. The last fact strikingly distinguishes them from the Wolffian bodies, as shewing that their sequestration of peculiar materials from the blood is only for *temporary or occasional* purposes.

"10. This metabolical action in regard of the circulation—this alternate withdrawing and rendering up again of certain particular materials, may be called the *common function* of the class; they belong to the offices of vegetative life, and are accessory to the circulatory system; they act on the blood occasionally, in respect of its chemical constitution." P. 85.

Mr. Simon, before giving his physiological conclusions, draws a verbal distinction between the terms "use" and "function," which are often employed synonymously: taking "function" to mean merely that *which an organ does,* viewed absolutely and alone,—taking "use" to denote this action viewed *in its relations to the entire system;* "function," the organ's *method of fulfilling* an use; "use," Nature's *application* of the function. Applying this (as it seems to us) just distinction to the instance before us, the author believes that he has shown the Functions of the Thymus gland, and next proceeds to unfold its use.

"The function of the Thymus may be stated in these words: by means of an apparatus strictly analogous to that of true glands, it secretes into a closed cavity certain particular elements of nutrition. Further, the secretion has been shewn to occur differently under different circumstances; viz.,

(1) "In most animals it occurs only temporarily. The secreted matter then presents itself in a *fluid form,* and is related to the universal material of nourishment, the liquor sanguinis, by the closest affinity of ultimate chemical composition.

(2) "In some animals, after discharging this temporary function, the gland gradually passes into the permanent exercise of a different, but analogous, act of assimilation, and manifests its secretion in the *solid form of fat.*" P. 86.

Mr. Simon further remarks:—

"By principles developed in our morphological analysis, we are enabled to explain the twofold difference observed in the secreted products of the gland,—that they are sometimes fluid and albuminous, sometimes solid and fatty. In the instance of the hybernators, the accumulation has reference to a state extending over many months: during half the year the nutritive material is being gathered into its receptacles, during the other half is becoming slowly exhausted: so gradual are these processes, that the ultimate organisation of the gland assumes a form expressive of *permanence;* namely, the cytoblasts undergo their extreme development, and the cells originating from them become filled with a more elaborated secretion than that of the temporary Thymus. In the other instance (that of the young animal) the functions of the gland are exercised amid conditions of hourly change: as the amount of ingesta, on the one hand,—as the breathing and muscular movements, on the other, may respectively be more, or less; so will the Thymus have at its disposal a larger, or smaller, surplus from the general expenditure,—so shew a richer, or a poorer, sinking-fund of nourishment. Since the circumstances, which render the Thymus an index of the general nutrition, vary from hour to hour, we are prepared to expect that the peculiarities of the ultimate organisation of the gland in young animals should be those of *im-permanence*." 87.

Knowing, then, that the Thymus presents an apparatus for the retention of nourishment in organic combination—knowing too that this combination is temporary, and is made by Nature (with varying degrees) so loose, that it may readily yield in proportion to the needs of the system, the author remarks, we still have the task before us of assigning an object to these contrivances. We must refer our readers to the work itself for the facts and arguments which lead him to the conclusion, that the THYMUS GLAND FULFILS ITS USE AS A SINKING-FUND OF NOURISHMENT IN THE SERVICE OF RESPIRATION.

" For—whereas the operations of the gland are essentially œconomical, and its loosely-combined products are ever held at the call of the system; whereas its presence is distinctly conterminous with the highest form of respiratory function; whereas its actions obviously pertain to periods having a common physiological peculiarity;—how can we avoid identifying its use with the exigencies of these periods and of that function? The nature of those exigencies has already been stated; and I can conceive no manner in which the functions of the Thymus may be applied to meet them, other than this,—that what the gland sequestrates from the circulation does, in gradually reverting thither, accomplish those chemical purposes in respect of respiration and temperature, which under other circumstances are fulfilled by the effete products of active animal tissues.

" In other words:—An object (probably the single essential object) of the respiratory process is the maintenance of an independent temperature for the animal. . . . In order to this end, the particles of the body are gradually yielded to the oxidising influence of the atmosphere; they slowly burn. . . . In the active tissues the rapidity of oxidation is in the ratio of exercise. . . . There are times and circumstances when these tissues waste infinitely little. . . . For such occasion how is oxidable matter provided? Surely by prior œconomy, by prior sequestration. . . . And this may occur in a two-fold manner; most usually in the form of fat the material is diffused throughout the body, filling its interstices and clothing its surface; more rarely it is gathered within a single organ, and retains the albuminous form in which it was first added to the blood. The requisite link for uniting these two instances is furnished by the fact, that under certain circumstances the second merges in the first; the Thymus deviates from its more special function—it passes into the general method of œconomical secretion—it fills its cavities with fat-cells—it becomes, indeed, a mere glanduliform collection of fat." P. 90.

On this theory, Mr. Simon believes that every fact that has come to his knowledge in the history of the Thymus admits of ready explanation;—the larger anatomy of the gland, and its minute structure; the chemistry of its products; its morphetical analogies; the laws regulating its presence and assigning its abrupt termination; the phenomena of its development and decrease; its invariable augmentation after birth; its occasional persistence through life; the evident proportioning of its functions to special and individual circumstances of nutrition and muscular activity. And while observing that his theory thus serves to combine, harmonize and explain the various facts which he has developed, he plainly shows that many of these facts are inflexibly opposed to the several other theories previously enumerated.

In an Appendix, there is a Note on the Pathology of the Thymus, a subject as yet very little investigated. We find nothing particularly deserving of notice, excepting an explanation of some enlargements of this gland, in accordance with the author's views of its use in the œconomy.

We here take leave of Mr. Simon's interesting and valuable work, and it only remains for us to record the favourable opinion we entertain of the author's talents and industry. Fully acquainted with and justly appreciating the labours of his predecessors, he has pursued his investigations in the highest department of physiological science in the legitimate spirit of induction, collecting and analysing facts, and advancing step by step in his inquiries, until, by observation of the phenomena and consideration of the laws of life, he has developed the functions and formed a theory of the use of the Thymus gland. We have endeavoured to present our readers with an analysis of his views, without attempting to discuss them. They must be carefully studied, and whether his opinions after examination be received or not, we venture to assert that his labours, as exhibited in this work, cannot fail to obtain for him a high reputation as an original inquirer and physiologist, and to secure an universal assent, that he has amply merited the distinguished honor which has been awarded him.

